# Bacterial biofilms in infective endocarditis: an in vitro model to investigate emerging technologies of antimicrobial cardiovascular device coatings

**DOI:** 10.1007/s00392-020-01669-y

**Published:** 2020-05-22

**Authors:** Alexander Lauten, Marc Martinović, Laura Kursawe, Judith Kikhney, Klaus Affeld, Ulrich Kertzscher, Volkmar Falk, Annette Moter

**Affiliations:** 1 Deutsches Zentrum für Herz-Kreislauf-Forschung (DZHK), Standort Berlin, Berlin, Germany; 2 Department of Cardiology, Charité, Universitaetsmedizin Berlin, Campus Benjamin Franklin, Hindenburgdamm 30, 12203 Berlin, Germany; 3 Department of General and Interventional Cardiology and Rhythmology, HELIOS Klinikum Erfurt, Erfurt, Germany; 4 Devie Medical GmbH, Bachstr. 18, 7743 Jena, Deutschland; 5 Biofilmcenter, Department of Microbiology, Infectious Diseases and Immunology, Charité, Universitaetsmedizin Berlin, Berlin, Germany; 6 Institute for Cardiovascular Computer-Assisted Medicine, Labor für Biofluidmechanik, Charité, Universitaetsmedizin Berlin, Berlin, Germany; 7 Department of Cardiothoracic and Vascular Surgery, German Heart Center Berlin, Berlin, Germany

**Keywords:** Infective endocarditis, In vitro model, Bioreactor, Staphylococcus epidermidis, Fluorescence in situ hybridization, Biofilm

## Abstract

**Objective:**

In spite of the progress in antimicrobial and surgical therapy, infective endocarditis (IE) is still associated with a high morbidity and mortality. IE is characterized by bacterial biofilms of the endocardium, especially of the aortic and mitral valve leading to their destruction. About one quarter of patients with formal surgery indication cannot undergo surgery. This group of patients needs further options of therapy, but due to a lack of models for IE prospects of research are low. Therefore, the purpose of this project was to establish an in vitro model of infective endocarditis to allow growth of bacterial biofilms on porcine aortic valves, serving as baseline for further research.

**Methods and results:**

A pulsatile two-chamber circulation model was constructed that kept native porcine aortic valves under sterile, physiologic hemodynamic and temperature conditions. To create biofilms on porcine aortic valves the system was inoculated with *Staphylococcus epidermidis* PIA 8400. Aortic roots were incubated in the model for increasing periods of time (24 h and 40 h) and bacterial titration (1.5 × 10^4^ CFU/mL and 1.5 × 10^5^ CFU/mL) with 5 L cardiac output per minute. After incubation, tissue sections were analysed by fluorescence in situ hybridization (FISH) for direct visualization of the biofilms. Pilot tests for biofilm growth showed monospecies colonization consisting of cocci with time- and inocula-dependent increase after 24 h and 40 h (*n* = 4). In *n* = 3 experiments for 24 h, with the same inocula, FISH visualized biofilms with ribosome-containing, and thus metabolic active cocci, tissue infiltration and similar colonization pattern as observed by the FISH in human IE heart valves infected by *S. epidermidis.*

**Conclusion:**

These results demonstrate the establishment of a novel in vitro model for bacterial biofilm growth on porcine aortic roots mimicking IE. The model will allow to identify predilection sites of valves for bacterial adhesion and biofilm growth and it may serve as baseline for further research on IE therapy and prevention, e.g. the development of antimicrobial transcatheter approaches to IE.

**Graphic abstract:**

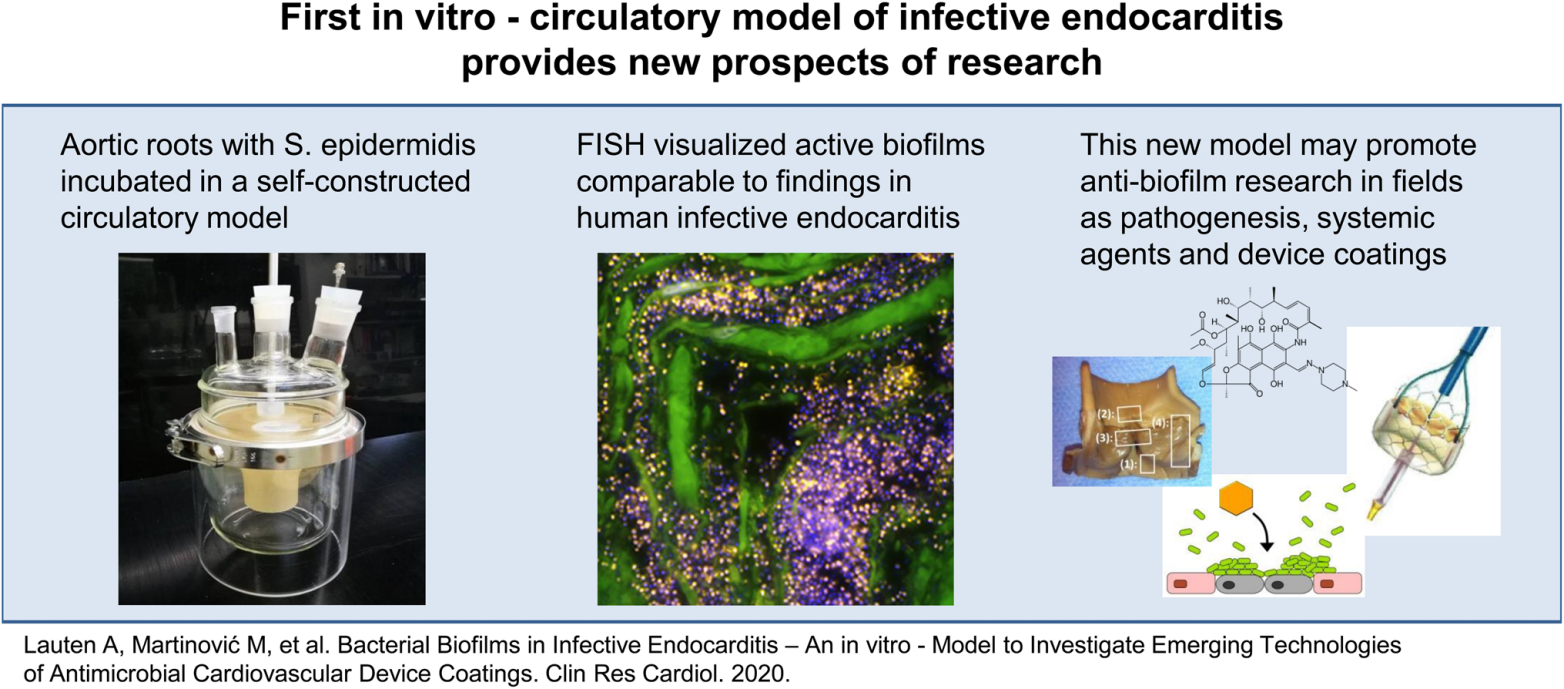

## Introduction

Despite recent advances in antimicrobial as well as surgical therapy, infective endocarditis (IE) remains a major clinical problem with mortality rates of 20–25% [2, 11, 28, 29]. As pharmacological and surgical treatment concepts are frequently ineffective, novel preventive and therapeutic treatment approaches are needed. However, prior to human application extensive preclinical evaluation of novel treatments in representative models of endocarditis is required.

When bacteria colonize endocardial tissue they form biofilms, which represent complex communities embedded into a matrix of secreted macromolecules [12]. The biofilm represents a protective environment for bacteria to tolerate systemic antibiotics and host phagocytic defenses. Different mechanisms may contribute to therapy failure, like poor penetration of many antibiotics, nutrient limitation, slow growth, low metabolic activity, and the formation of persister cells [10, 14, 18, 23, 32, 33]. The resulting recalcitrance towards antibiotic treatment poses a significant problem in endocarditis.

Although animal models (particularly in rabbits) are commonly used to evaluate the efficacy of antimicrobial agents in bacterial endocarditis, these models are costly, difficult to standardize and are frequently not suitable for the evaluation of human cardiovascular implants [17, 37]. Therefore, in vitro models of bacterial endocarditis may present a more predictable method to generate bacterial growth in a timely and reproducible manner, also avoiding ethical considerations.

So far, the development of bacterial biofilms on native aortic valves has not been studied in ex vivo systems. A better knowledge of the mechanisms involved in biomaterial infection is, therefore, essential. Herein, we present a novel pulsatile in vitro model of bacterial endocarditis, allowing the growth of bacterial biofilms on porcine aortic roots in toto under physiologic hemodynamic conditions.

## Methods

### Description of the bioreactor endocarditis model

**Fig. 1:**
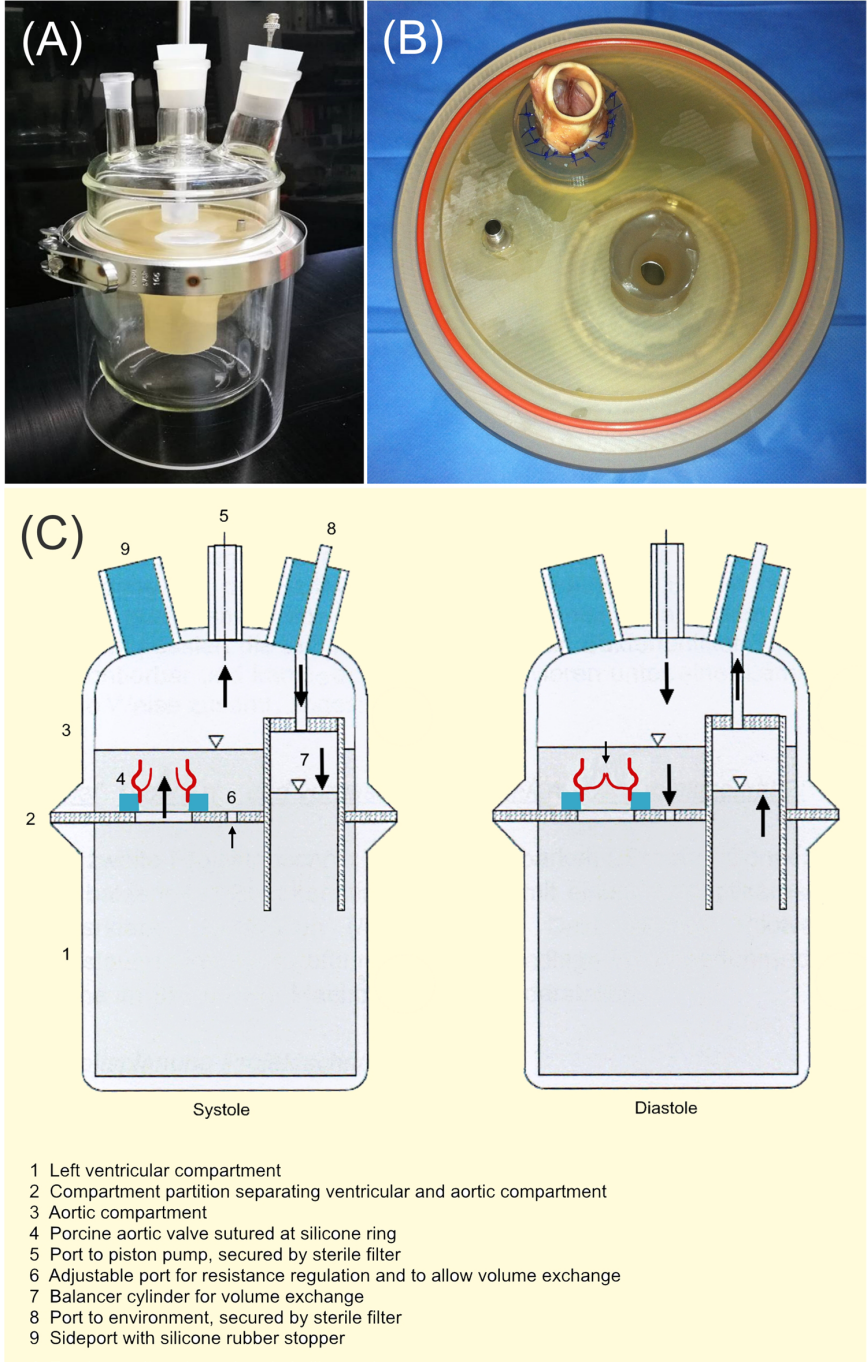
Endocarditis bioreactor model. Pulsatile circulatory model implementing physiologic temperature and in vivo pulsatile flow conditions. Porcine valves are positioned between the aortic and ventricular compartment. The model allows bacterial growth on the heart valves and reproduces hydrodynamic factors and physical shear stress comparable to biofilm formation conditions in humans in vivo

A 1500 mL circulatory bioreactor model (LB-Engineering, Berlin, Germany) was designed to allow bacterial colonialization and biofilm growth on native porcine heart valves, prosthetic tissue or mechanical heart valves and cardiovascular implants (Fig. 1). The model aims for physiologic temperature and human in vivo pulsatile flow conditions to mimic bacterial growth in IE and reproduce hydrodynamic factors and physical shear stress which are important factors involved in biofilm formation in vivo. Tryptone soy broth without dextrose (TSB) culture medium (Sigma Aldrich, St. Louis, USA) was used as blood substitute. The flow in the bioreactor was generated with pulsatile air pulses generated by a piston pump (Harvard Apparatus, MA, USA). The air inside the bioreactor was protected from ambient air by sterile breathing system filters (Pall Corporation, NY, USA). To guarantee physiologic temperature conditions the system was placed in an incubator cabinet at 37 °C.

As stroke volume and heart frequency of the pump are freely adjustable in the range of 0–100 mL and 0–100 bpm, the pump generates a physiologic, pulsatile flow through the aortic valve. If the piston moves towards the aortic valve, the valve will close. If it moves back, the valve will open again. Before the pump was started, an air volume of 150 mL was removed from aortic compartment to realize a lower gauge height in the balancer cylinder than in aortic compartment to ensure that no TSB could reach the sterile air filter above. For this purpose, a cannula was pierced into the free silicone rubber stopper till it reached aortic compartment’s lumen and a sterile three-way valve (BRAUN, Melsungen, Germany) and 50 mL syringe (BRAUN, Melsungen, Germany) were mounted to remove air and thus adjust gauge height in the balancer cylinder.

To test the bioreactor’s microbial isolation towards the environment, sterility tests (*n* = 3) were held. The bioreactor was filled with 1500 mL TSB and ran for 24 h as mock model without aortic valve inside at 37 °C.

In addition sterility of porcine aortic valves after its fixation was tested by FISH analysis (*n* = 2) and incubation in TSB Bouillon for 48 h (*n* = 3).

### Tissue preparation

Aortic valves harvested from porcine hearts were prepared as previously described [21, 22]. For use in the bioreactor model, valves were mounted manually on a silicone ring by means of a simple interrupted suture (3-0 Prolene, Ethicon, NJ, USA). All devices were stored at least 7 days in 0.6% glutaraldehyde (GA) (Carl Roth, Karlsruhe, Germany) and rinsed for 24 h in phosphate buffered saline (PBS) immediately before use.

### Bacterial strain and biofilm growth

To determine optimal conditions for biofilm creation, system pilot tests (*n* = 4) with different bacterial inocula and test duration were performed. First the bioreactor was autoclaved, filled with 1500 mL TSB and the aortic valve was inserted. Then the system was inoculated with 1 mL or 10 mL of *Staphylococcus epidermidis* PIA 8400 dissolved in TSB [24]. This strain is a well described biofilm producer originally isolated from a patient. All inocula were calibrated to an optical density (OD) of 0, 1. The number of viable bacteria was exemplarily confirmed by plating and counting the colony forming units (CFU). The 1 mL inoculum was equivalent to ca. 1.5 × 10^4^ CFU/mL in the system, the 10 ml inoculum corresponded to 1.5 × 10^5^ CFU/mL. Tests ran at 37 °C for 24 h or 40 h with 5 L cardiac output per minute. To investigate the reproducibility of the created biofilms repeated trials (*n* = 3) with 1.5 × 10^5^ CFU/mL and 24 h duration were performed.

### Molecular analysis

Bacterial DNA was extracted from tissue sections with a commercially available specimen preparation kit (Amplicor, Roche Molecular Systems Inc, Branchburg, NJ, USA). Broad-range PCR amplification and sequencing of part of the 16S rRNA-gene were performed as described [8, 15]. Resulting sequences were compared to all the currently available data from public databases using the SmartGene platform (SmartGene, Lausanne, Switzerland).

### Tissue processing, fluorescence in situ hybridization (FISH) and microscopic evaluation

**Fig. 2:**
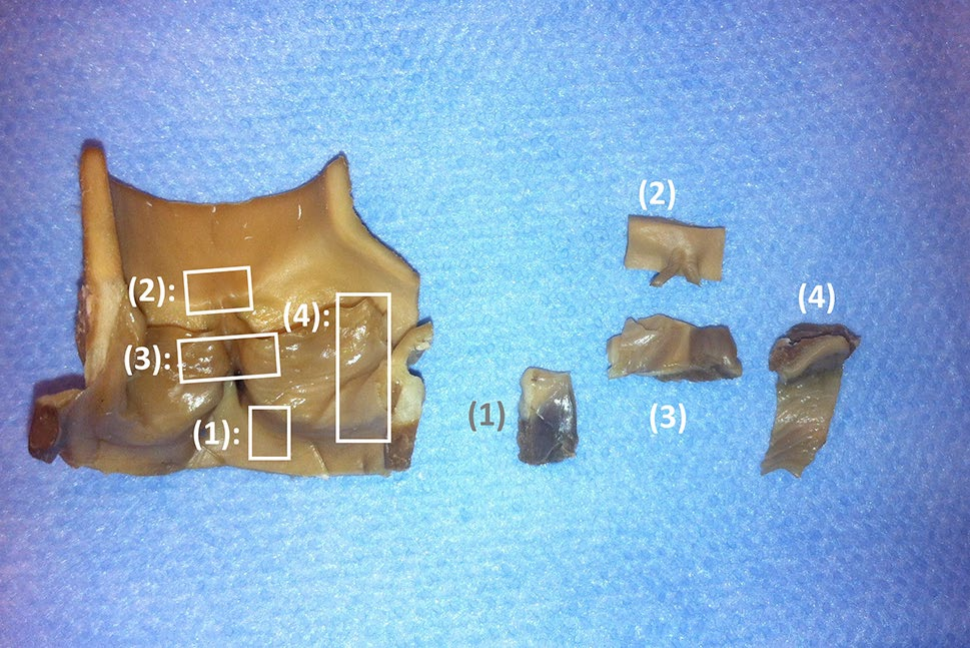
Localization sites of harvested samples after biofilm growth. (1) inter leaflet triangle, (2) commissure, (3) lateral leaflet cross-section, (4) middle leaflet cross-section

Valves were aseptically removed from the reactor and tissue samples were harvested from four different sections of the endocardium (Fig. 2) [5]. For each trial all four samples were analysed. The samples were fixed in FISH fixation solution and embedded in cold polymerizing resin Technovit 8100 (Kulzer, Wehrheim, Germany) according to the manufacturer’s instructions. Prior to embedding, the specimens were incubated overnight in PBS containing 6.8% (w/v) sucrose, dehydrated in acetone for 1 h and infiltrated with methacrylate solution [27].

FISH analysis was carried out on 2 µm tissue sections of each sample as described previously [27]. Tissue sections were hybridized with a hybridization buffer containing the nucleic acid stain 4′,6-diamidino-2-phenylindole (DAPI) and the FISH probes EUB338 (detecting most bacterial species), labelled at the 5′ end with the fluorescent indocarbocyanine dye Cy3 and non-EUB338 labelled with Cy5, to rule out unspecific probe binding. Furthermore, FISH probes which are STAPHY specific for *Staphylococcus* spp. labelled with FITC and SAU specific for *Staphylococcus aureus* labelled in Cy3 were applied in the selected samples to confirm the monospecies colonization of the heart valve [1, 19, 36].

Microscopic evaluation of the heart valves and biofilms was carried out with an AxioImager Z2 epifluorescence microscope (Zeiss, Jena, Germany) with narrow band filter sets (AHF Analysentechnik, Tübingen, Germany) and the ZEN Blue software (Zeiss, Jena, Germany). All tests were investigated qualitatively. In addition bacterial colonization of the three reproducibility tests was manually measured pertaining to thickness and invasiveness of the biofilms.

## Results

### Sterility tests and negative controls

After 24 h incubation with sterile TSB medium at 37 °C in all *n* = 3 sterility tests, no bacterial growth was observed in the system. Plating of the medium on agar plates remained negative.

FISH analysis of *n* = 2 porcine aortic valves after 7 days incubation in 0.6% GA revealed no active microorganisms at the valve and after incubation of *n* = 3 in TSB Bouillon for 48 h no microbial growth was observed in Bouillon as well as after plating the Bouillon on agar plates.

### Bacterial colonization and biofilm formation on porcine heart valves

For all seven valves tested with bacterial inoculation of the medium, macroscopic evaluation demonstrated intact valve structure and function after 24 h and 40 h of incubation. No macroscopic bacterial vegetations were observed.

Microscopic evaluation of *n* = 4 pilot tests by FISH revealed the presence of monospecies staphylococcal colonization. It showed all different bacterial formations ranging from single adhered cocci to multilayered biofilms. All biofilms featured ribosome-containing, FISH-positive bacteria thus corresponding to metabolic active bacteria [30]. In comparison biofilms of these pilot tests had varying degrees of thickness and invasiveness associated with the inoculum and less with the duration of incubation. The pilot test with higher bacterial inoculum (1.5 × 10^5^ CFU/mL) and shorter duration (24 h) showed best efficacy in terms of ratio of biofilm thickness to test duration.

**Fig. 3:**
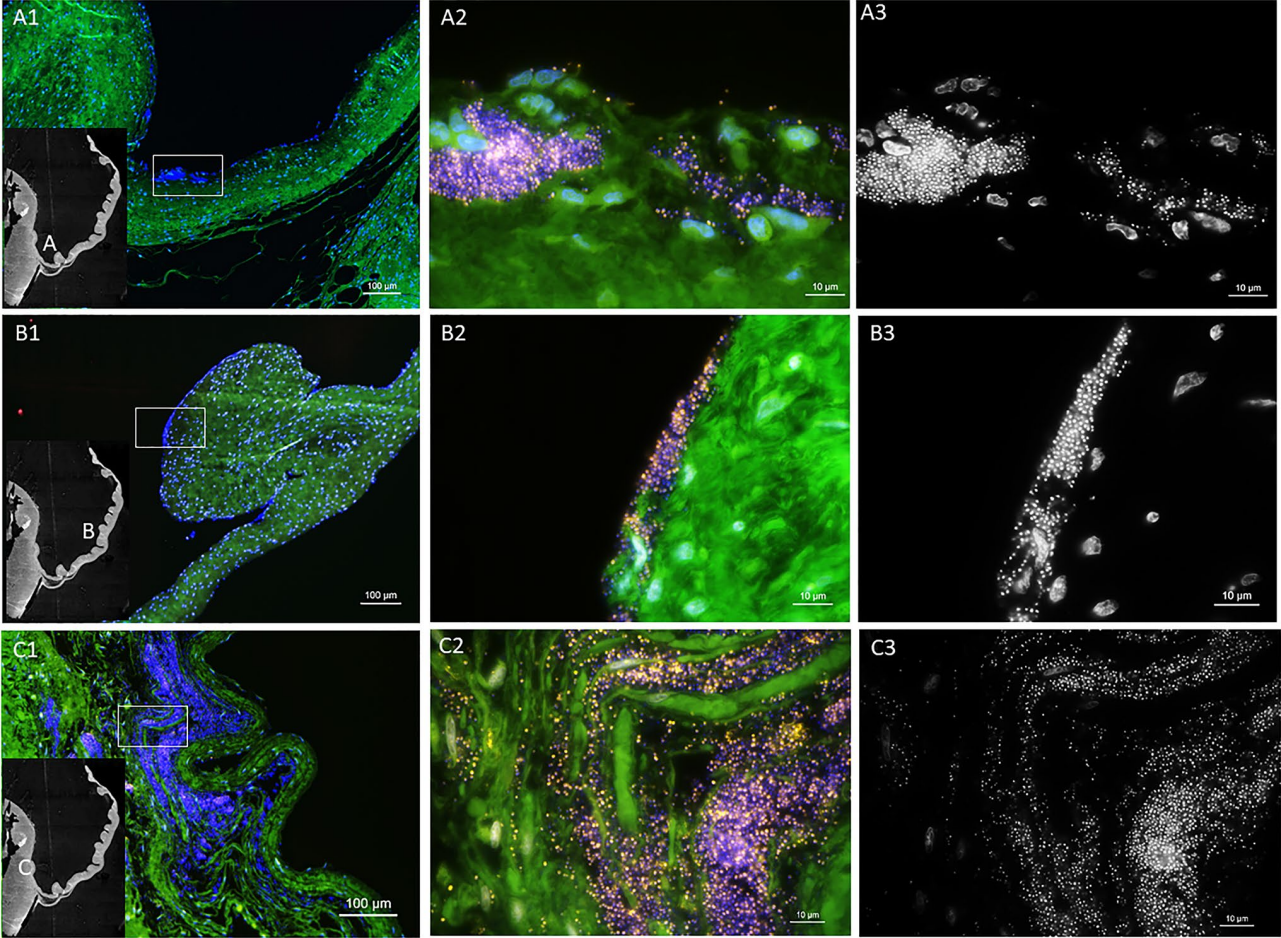
FISH of tissue sections from ventricular surface of aortic valve leaflets. FISH analysis of tissue sections from ventricular surface of aortic valve leaflets incubated with *S. epidermidis* for 24 h in the bioreactor. FISH was performed using the pan-bacterial probe EUB338-Cy3 (yellow) and nucleic-acid-specific DAPI stain. Unspecific binding was excluded, using the nonsense probe NON338 (data not shown). (A1) A pattern of the aortic valve leaflet is included in the overview of location A at the beginning of the leaflet (bar = 100 µm). (A2) Higher resolution of the inset in A1. Overlay of all channels shows a small biofilm with EUB338 positive bacteria (yellow) (bar = 10 µm). (A3) Identical microscopic field of the DAPI channel in black (bar = 10 µm). (B1) Overview of location B at the middle of the leaflet (bar = 100 µm). (B2) At higher magnification FISH shows an active biofilm at the border of the tissue (bar = 10 µm). (B3) DAPI staining in black (bar = 10 µm). (C1): Overview of the location C in the endocardium (bar = 100 µm). (C2) Higher magnification of inset C showing massive infiltrating biofilms (bar = 10 µm). (C3) DAPI channel in black (bar = 10 µm)

FISH analysis of *n* = 3 repeated assays with 1.5 × 10^5^ CFU/mL titration and 24 h duration showed the same staphylococcal colonization pattern as seen in the pilot tests. The four samples per trial compared to each other confirmed the findings from the pilot tests, that bacterial colonization was focused on the aortic leaflet itself, whereupon the leaflet’s free margin and its base seemed to be particularly colonized. All three trials showed a similar colonization pattern on the leaflets containing biofilms with a thickness ranging from 2 to 25 µm on the leaflet’s luminal surface and invasive biofilms spreading underneath the endothelium reaching Stratum myoelasticum or even Tela subendocardialis (Fig. 3). Accordingly invasive biofilms reached a maximum invasiveness of 400 µm from the surface with a thickness ranging from 4 to 100 µm.

Two PCRs performed at random showed the inoculated *S. epidermidis* as a pathogen of the biofilm with a matching genotype of 100% sequence identity over 509 bp and 508 bp, respectively. Also specific FISH, with the probes STAPH and SAU, performed in two heart valve samples confirmed the FISH-positive cocci as *Staphylococcus* species and excluded contamination with *Staphylococcus aureus* species.

**Fig. 4:**
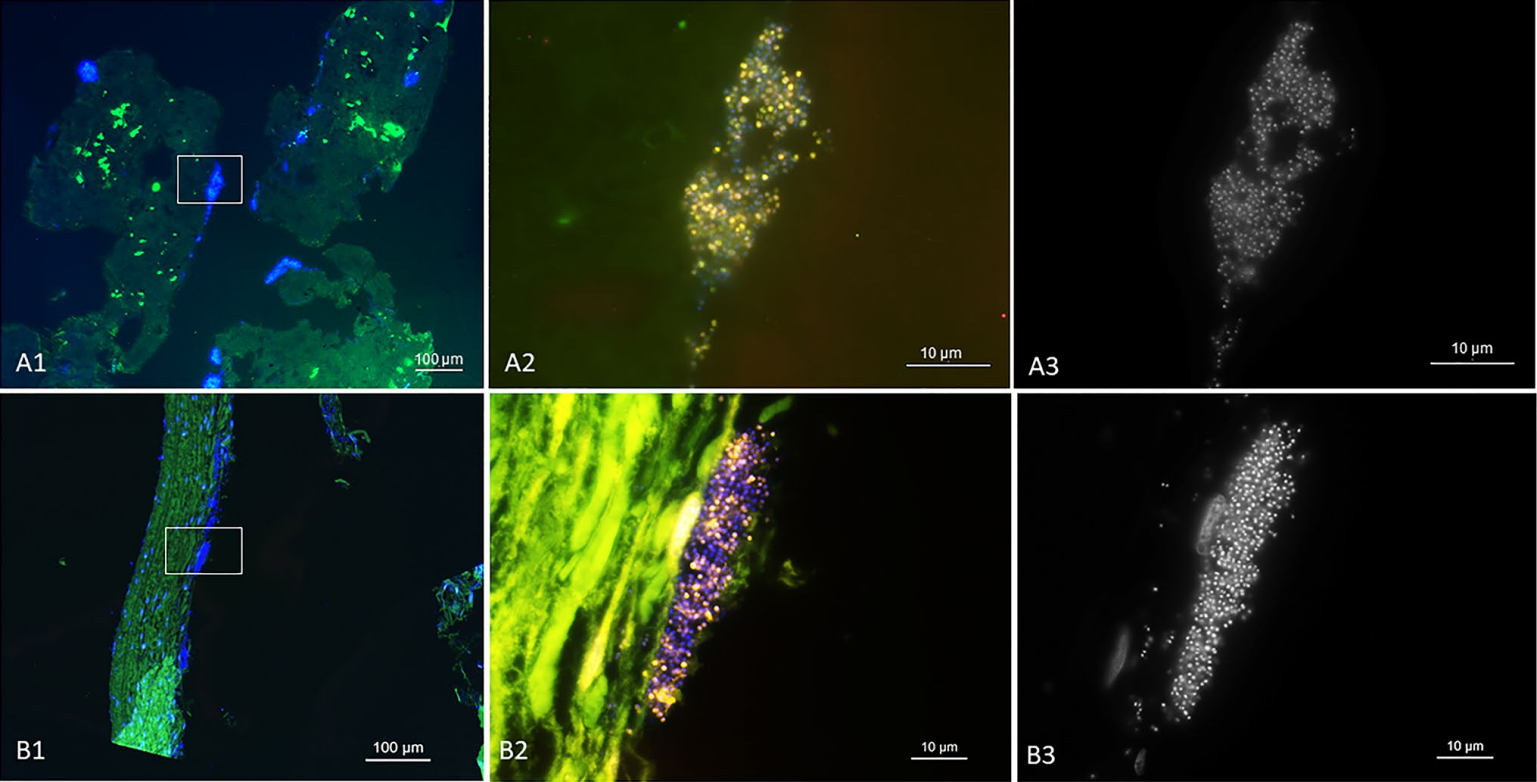
In vitro colonization corresponds to clinical human IE findings. FISH analysis of a human heart valve from a patient with *S. epidermidis* IE (**a**) compared to a porcine heart valve infected by *S. epidermidis* in the in vitro endocarditis bioreactor for 24 h (**b**). FISH using the pan-bacterial probe EUB338-Cy3 (yellow) and unspecific nucleic acid stain DAPI. Unspecific binding was excluded, using the nonsense probe NON338. (A1) Overview of the human heart valve tissue (bar = 100 µm). (A2) Higher magnification of inset (A1) showing an overlay of all channels, reveals a biofilm with EUB338-Cy3 positive cells (bar = 10 µm). (B1) Overview of the porcine leaflet tissue (bar = 100 µm). (B2) At higher magnification biofilms in the tissue appeared with strong EUB338-Cy3 FISH signals (bar = 10 µm). (A3, B3) Note the similar pattern in black and white images of the single channel DAPI, highlighting the biofilm formation (bar = 10 µm)

Comparison of the FISH results with the in vitro-created biofilms showed a good comparability to in vivo-grown biofilms of *S. epidermidis* in human heart valves from IE patients (Fig. 4).

## Discussion

IE is a therapeutic challenge to routine clinical practice as current treatment approaches are frequently ineffective. This is due to multiple factors, such as the limited bactericidal effect of systemic antibiotic drug treatment resulting from the altered metabolic state of bacteria in the microenvironment of the bacterial biofilm.

Suitable models of bacterial growth on the endocardial surface of heart valves and cardiovascular implants are required to study the development and structure of bacterial biofilms and investigate novel preventive or therapeutic strategies to treat IE.

Animal models of bacterial endocarditis are well established and serve as valuable tools to investigate novel therapeutic approaches before transferring them to human treatment. Particularly the rabbit model is frequently applied to evaluate the efficacy of new antibiotic drugs and therapeutic concepts of endocarditis prior to human use.

However, apart from ethical considerations and the associated costs, in vivo animal models have limitations, such as the limited predictability and reproducibility of in vivo biofilm development and as well as the variability of pharmacokinetics of antimicrobial agents in vivo. Further, models in small animals such as rabbits are not suitable for the evaluation of human cardiovascular implants. Several in vitro biofilm models have been reported in the literature, but to our knowledge no model has been developed so far to investigate bacterial biofilm growth developing on native heart valves and implants under physiologic hemodynamic conditions [4, 9, 20, 25, 38].

The model described here is the first to allow in vitro biofilm development on porcine native aortic valves in toto, including the specific pathophysiological steps leading to bacterial growth and infiltration of the endocardium and adjacent myocardium, such as bacterial adhesion and colonization of the valve endothelium, bacterial replication, the production of extracellular polymers, biofilm formation and invasion of organ tissue. The here presented data showed that the model is capable of performing repeated trials to create a microbial colonization pattern on native aortic valves equivalent to colonization patterns observed in human IE and consequently simulating infective endocarditis in vitro. The finding that predominantly the leaflets themselves were affected by bacterial colonization corresponds to observations made before in human IE [13]. This in vitro model of IE may thus contribute in the future to a better understanding of the pathogenesis and pathophysiology of the infection and provides the foundation for further research, thus helping to improve the therapeutic and prophylactic regimens of IE in humans. As current research estimates that up to 80% of human infections are related to biofilms, new models like the one presented here are needed to promote the growing field of research on pathogenesis of biofilms and innovative anti-biofilm strategies [3].

Recent data show, that about one quarter of patients with formal surgery indication cannot undergo surgery and patients with prosthetic valve endocarditis, especially with transcatheter aortic valve implantation (TAVI) endocarditis, have an increased mortality [6, 7, 16, 31, 34]. Both, therapeutic and preventive antimicrobial coatings on prosthetic heart valves, may help to improve the poor prognosis of those patients.

### Limitations

In vital organisms, platelets and fibrin are involved in the establishment of a bacterial vegetation. Following the endocardial injury and focal adherence of platelets and fibrin, the platelet–fibrin nidus becomes secondarily infected by microorganisms [6, 8, 10, 26]. Limitations of the here described in vitro endocarditis model are the lack of host defence mechanisms, including the local inflammatory response with adhesion and migration of immune cells and complement activation. TSB culture media were used to replace blood and aortic roots fixed in glutaraldehyde without vital endothelium. Additionally, trial duration in the in vitro model probably differs from the length of aortic valve colonization in vivo and the used TSB culture media features a different nutrition and viscosity than human blood. To get even closer to the situation in vivo, regarding the further development of the model it could be considered to use anticoagulated blood as the medium instead of TSB, but in all probability this would exponentially increase the challenges for a reproducible model [35, 39].

## Conclusion

In conclusion, the model described herein reproduces the pathogenesis and pathophysiology of bacterial biofilm formation on native heart valves under physiologic flow conditions and may thus promote further research on the development of IE. Despite the limitations of an ex vivo model, the model can be central to the development and evaluation of novel therapeutic and preventive strategies against infective endocarditis. For this purpose the model’s broad potential reaches e.g. from testing of systemic antimicrobial agents to the development of antimicrobial device coatings.
